# Exploring the Anti-Hypoxaemia Effect of Hydromethylthionine: A Prospective Study of Phase 3 Clinical Trial Participants

**DOI:** 10.3390/ijms241813747

**Published:** 2023-09-06

**Authors:** Mohammad Arastoo, Michael P. Mazanetz, Sonya Miller, Helen Shiells, Claire Hull, Keith Robinson, John M. D. Storey, Charles R. Harrington, Claude M. Wischik

**Affiliations:** 1 School of Medicine, Medical Sciences and Nutrition, University of Aberdeen, Aberdeen AB25 2ZP, UK; s11ma7@abdn.ac.uk (M.A.); c.harrington@abdn.ac.uk (C.R.H.); 2 Scottish Biologics Facility, School of Medicine, Medical Sciences and Nutrition, University of Aberdeen, Aberdeen AB25 2ZP, UK; 3 NovaData Solutions Ltd., 15 Monreith Rd, Newlands, Glasgow G43 2NX, UK; mikem@novadatasolutions.co.uk; 4 Department of Chemistry, University of Aberdeen, Aberdeen AB24 3UE, UK; j.storey@abdn.ac.uk; 5 TauRx Therapeutics Ltd., 395 King Street, Aberdeen AB24 3FX, UK; s.miller@taurx.com (S.M.); h.shiells@taurx.com (H.S.); c.hull@taurx.com (C.H.); 6 Syneos Health, LLC, Morrisville, NC 27560, USA; keith.robinson@syneoshealth.com

**Keywords:** hydromethylthionine, hypoxaemia, anti-hypoxaemia, COVID-19, SARS-CoV-2

## Abstract

Methylthioninium chloride (MTC) is a standard treatment for methaemoglobinaemia. A preparation of reduced MTC has been reported to increase blood oxygen saturation (SpO_2_) and lower respiratory rates in patients with severe COVID-19. We have developed a stable form of reduced methylthionine (hydromethylthionine-mesylate, HMTM) having a benign safety profile in two Phase 3 trials in Alzheimer’s disease. The aim of this prospective study was to determine the effects of oral HMTM on SpO_2_ and methaemoglobin (metHb) levels in a cohort of patients with mild hypoxaemia not due to COVID-19. Eighteen participants randomised to a single dose of 4, 75, 100 or 125 mg doses of HMTM had SpO_2_ levels below 94% at baseline. Patients were routinely monitored by pulse oximetry after 4 h, and after 2 and 6 weeks of twice daily dosing. Significant ~3% increases in SpO_2_ occurred within 4 h and were sustained over 2 and 6 weeks with no dose differences. There were small dose-dependent increases (0.060–0.162%) in metHb levels over 2 to 6 weeks. Minimum-energy computational chemistry revealed that HMT can bind within 2.10 Å of heme iron by donating a pair of electrons from the central nitrogen of HMT to *d* orbitals of heme iron, but with lower affinity than oxygen. In conclusion, HMTM can increase SpO_2_ without reducing metHb by acting as a strong displaceable field ligand for heme iron. We hypothesise that this facilitates a transition from the low oxygen affinity T-state of heme to the higher affinity R-state. HMTM has potential as an adjunctive treatment for hypoxaemia.

## 1. Introduction

Hypoxaemia is characterised by a blood oxygen saturation (SpO_2_) less than 95% due to a variety of conditions including asthma, anaemia, pneumonia, chronic obstructive pulmonary disease, acute respiratory distress syndrome, and pulmonary fibrosis [1]. There is currently no specific pharmacologic intervention for the treatment of hypoxaemia. A compound which improves SpO_2_ levels safely and reversibly has the potential to provide an adjunct to available treatments.

Methylthioninium chloride (MTC, methylene blue) is a standard treatment for methaemoglobinaemia [2]. The methylthionine (MT) moiety can exist in the oxidised MT^+^ form (in MTC) and in the reduced hydromethylthionine (HMT) form [3]. MT^+^ needs to first be converted to HMT to permit absorption, distribution and uptake into cells [4,5]. A stabilised form of HMT (hydromethylthionine mesylate, HMTM) was developed to permit the direct administration of HMT for the treatment of Alzheimer’s disease (AD) [3,4,6,7,8] because HMT is a potent tau aggregation inhibitor [9].

It is well established that MT is able to interact with the heme component of haemoglobin [2,10]. In methaemoglobinaemia, heme iron is in the ferric (Fe^3+^) rather than the ferrous (Fe^2+^) form which has reduced the ability of haemoglobin to carry oxygen [10]. HMT is the active species at the heme site of action where it facilitates the transfer of an electron from HMT to Fe^3+^, reducing it to Fe^2+^ and oxidising HMT to MT^+^ in the process. The continuing regeneration of HMT via ongoing red cell glycolysis permits the restoration of normal oxygen-carrying capacity [11,12].

There has been increasing recent interest in MTC as a potential treatment for COVID-19 [13,14,15]. COVID-19 is associated with hypoxaemia due both to lung damage and methaemoglobinaemia, limiting the oxygen-carrying capacity of haemoglobin [16]. The potential benefit of HMT in the treatment of critically ill COVID-19 patients was recently reported [13,17]. In the first report, HMT (given as a syrup containing MTC, ascorbic acid as a reducing agent and N-acetyl cysteine as an expectorant) was administered to five patients who had been admitted to intensive care and who had failed to respond to other forms of treatment. The authors reported a comparison of the four surviving subjects before and after receiving HMT. Their mean oxygen saturation (SpO_2_) improved from 79% prior to treatment to 92% and methaemoglobin (metHb) levels reduced from 15% to 5%. In a more recent report, outcomes in 40 COVID-19 patients treated with the same MTC mixture in addition to standard of care were compared with 40 COVID-19 patients receiving only standard of care. In this study, the SpO_2_ levels improved from 80.0% pre-treatment to 88.9% and the respiratory rate decreased from 34.4% to 22.7% following 5 days of treatment with the MTC mixture at doses equivalent to 91–138 mg/HMT per day.

Although the reports in COVID-19 patients support further investigations, they do not permit the relative contributions of HMT, ascorbic acid and N-acetyl cysteine to be distinguished. They also do not permit determination of whether the effects on SpO_2_ are linked to a reduction in metHb. Finally, they do not permit the determination of whether the effects observed are specific to COVID-19 or whether they have a more general relevance for the treatment of hypoxaemia due to other causes. Because of the broader potential clinical utility of a specific anti-hypoxaemia drug, we undertook a preliminary investigation of the effects of HMTM treatment on SpO_2_ and metHb levels in patients participating in either of two Phase 3 clinical trials in AD [7,8] in which metHb and SpO_2_ levels were monitored routinely using pulse oximetry.

## 2. Results

### 2.1. Population Characteristics at Baseline

Data were available for 18 subjects from both AD studies who had oxygen saturation <94% at baseline (the lower limit of the normal range is 95%). Their demographic characteristics are summarised in Table 1. The mean age was 76.1 years, ranging from 70 to 80 years, with more females (61%) than males (39%).

Using medical history available from patient Case Report Forms, hypoxaemia in these subjects was found to be associated with a variety of underlying respiratory or other conditions of varying degrees of severity which could plausibly have contributed to chronic hypoxaemia (summarised in Table 2). These include sleep apnoea, insomnia, asbestosis, oedema, asthma, bronchitis, allergies, angioedema, pneumonia, acute myocardial infarction/hypertension, coronary artery disease with angioplasty and stent insertion, transient ischaemic attacks (TIA), hypothyroidism, diabetes, syncope, tachycardia and sepsis. No predisposing clinical history factors could be identified in three of the subjects.

### 2.2. Prospective Clinical Study of Peripheral Oxygen Saturation and Methaemoglobinaemia in AD Patients Treated with HMTM

**Figure 1:**
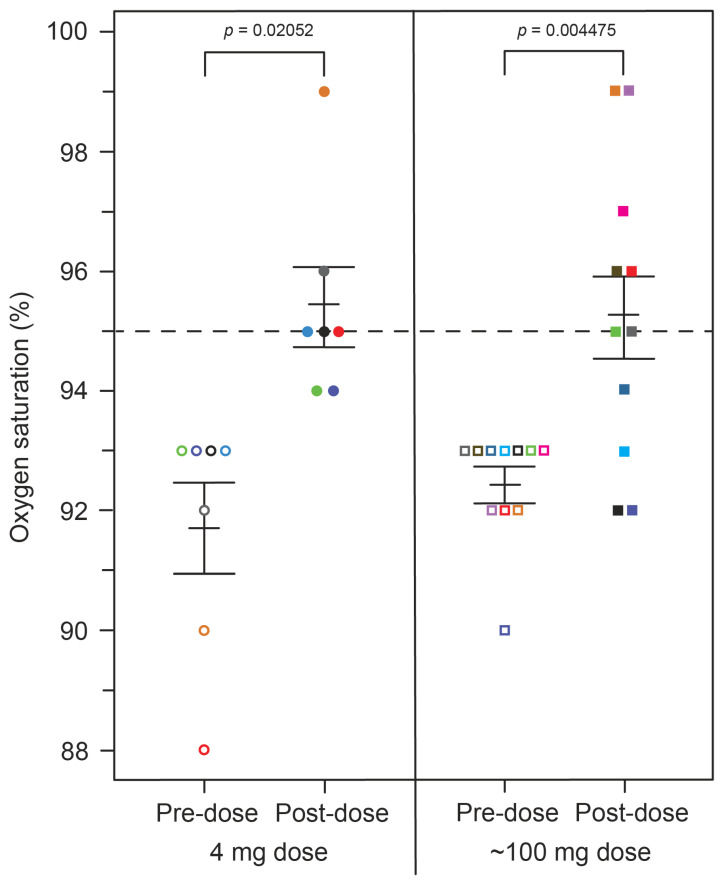
Changes in oxygen saturation levels in patients receiving HMTM, comparing pre-dose and after four hours (post-dose) following administration of single doses of HMTM at 4 mg and high doses (75/100/125 mg indicated as the mean dose, 100 mg). Data represent mean (%) ± S.E. The dashed line indicates a healthy oxygen saturation level of 95%. Open circles and squares correspond to subjects in the 4 mg and ~100 mg dose groups before dosing, respectively. Closed circles and squares represent subjects matched by colour after dosing. Colour coding of subjects also corresponds to data shown in Figure 4.

**Figure 2:**
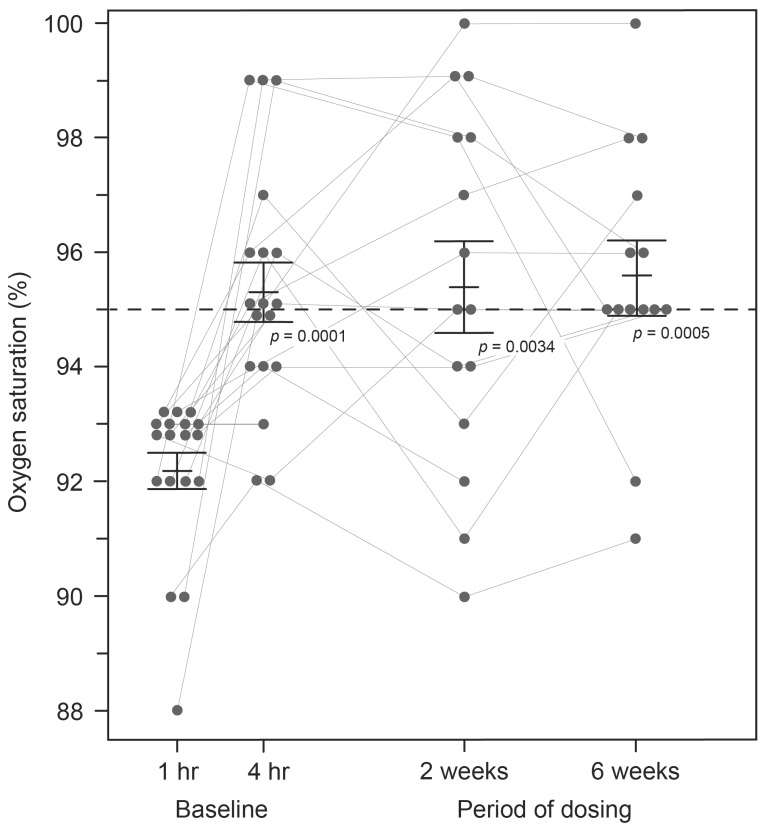
Changes in oxygen saturation levels in subjects receiving HMTM over a 6-week period. Pooled data for all doses represent mean (%) ± S.E. Statistical analysis relating to this graph is contained in Table 4.

SpO_2_ levels were compared pre-dose and after four hours in the clinic following the administration of a single dose of HMTM at 4 mg or doses of 75, 100 or 125 mg (summarised as the mean, 100 mg, for the high doses; Figure 1). As can be seen in Figure 1, HMTM at a dose of 4 mg substantially increased mean blood oxygen saturation levels. Mean oxygen saturation in the group with baseline SpO_2_ levels below 94% was 91.71%. Four hours after receiving a 4 mg dose of HMTM, the mean SpO_2_ level was increased to 95.43% (+3.72%, *p* = 0.0205; Table 3, Figure 1). Likewise, following the administration of high-dose HMTM, oxygen saturation levels increased from 92.45% at baseline to 95.27% after four hours (+2.82%, *p* = 0.0045; Table 3, Figure 1). Therefore, HMTM is able to increase blood oxygen saturation within 4 h across a broad range of doses with no discernible dose-dependent differences. As can be seen from Figure 2 and Table 4, the effect is stable over 6 weeks at 8 mg/day and 150–250 mg/day, and the differences with respect to baseline were statistically significant at week 2 (+3.17%, *p* = 0.0034) and week 6 (+3.23%, *p* = 0.0005). Therefore, HMTM produces a rapid improvement in oxygen saturation at single doses in the range 4–125 mg, and this is sustained over 6 weeks at doses in the range 8–250 mg/day.

**Figure 3:**
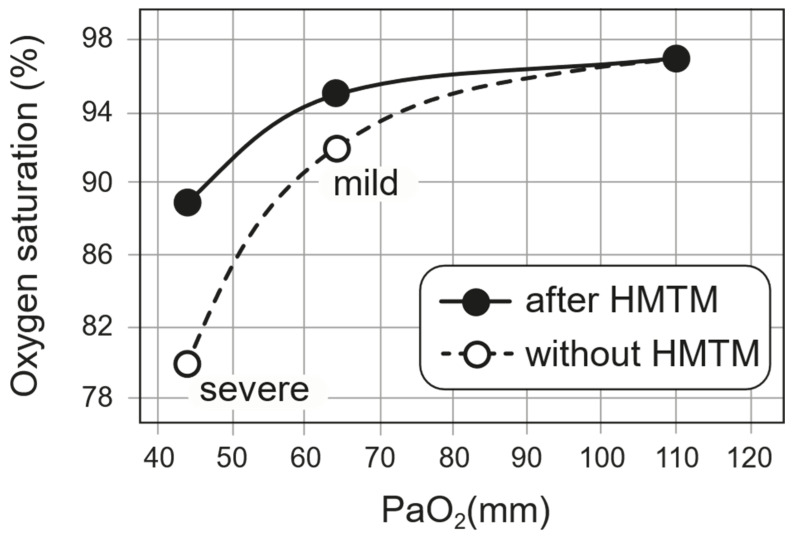
The implied effect of HMTM treatment on the oxygen–haemoglobin dissociation curve as described in the text. Data for patients with mild hypoxaemia came from subjects treated with HMTM. Data for patients with severe hypoxaemia came from results reported by Hamidi-Alamdari et al. (2021) [17] in which a preparation of reduced MTC was used to deliver HMT orally.

The implied effect of treatment on the oxygen–haemoglobin dissociation curve is shown in Figure 3. We have used a standard dissociation curve to estimate the corresponding PaO_2_ value at baseline. It can be seen that the effect of HMTM treatment in raising the SpO_2_ value corresponds to an implied left-shift in the oxygen–haemoglobin dissociation curve. The cases in which HMTM treatment were used had relatively mild hypoxaemia (Figure 3, ‘mild’). For more severe cases (Figure 3, ‘severe’), we have used the same approach to infer the implied effect on oxygen–haemoglobin dissociation in patients receiving the reduced MTC formulation reported by Hamidi-Alamdari et al. (2021) [17] using the untreated population as a basis for comparison. As can be seen, the implied effect of treatment with reduced MTC is similar. Considering the available SpO_2_ data for severe hypoxaemia, there was a ~10% improvement following treatment with reduced MTC. Assuming that the normal P50 is 25 mmHg, we could estimate that this improvement might correspond to a P50 of 27.5 mmHg as an approximation of the improved oxygen-binding affinity in the presence of HMTM. Alamdari et al. (2021) [17] reported a reduction in respiratory rate from 34.4 ± 5.5 to 22.7 ± 6.7. This reduction in respiratory rate reflects the left-shift in the oxygen–haemoglobin dissociation curve which gives better oxygenation at a given saturation. The reduction in respiratory rate would also be expected to decrease hypercapnia and associated acidosis.

**Figure 4:**
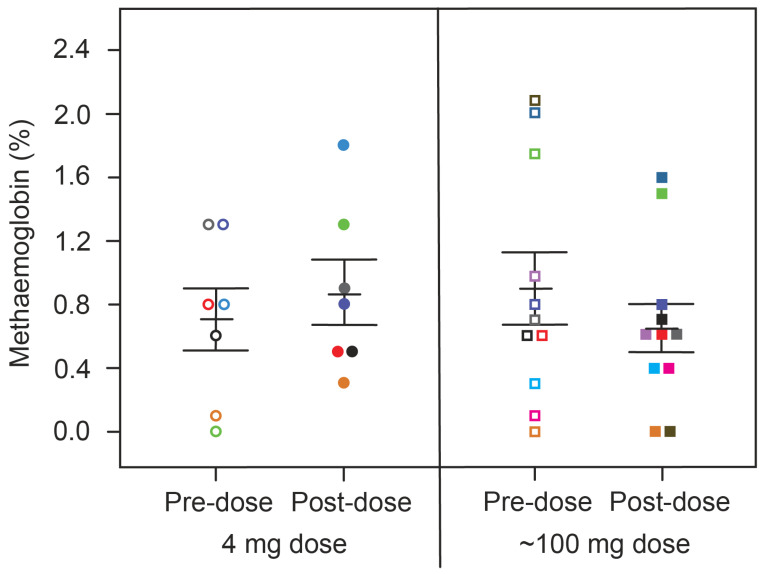
Changes in metHb levels in patients receiving HMTM, comparing pre-dose and after four-hours (post-dose) following administration of single doses of HMTM at 4 mg and high doses (75/100/125 mg indicated as the mean dose, 100 mg). Data represent mean (%) ± S.E., with *p* = 0.5472 and *p* = 0.2390 in the 4 mg dose group and ~100 mg dose group, respectively. Colour coding matches that for subjects in Figure 1.

**Figure 5:**
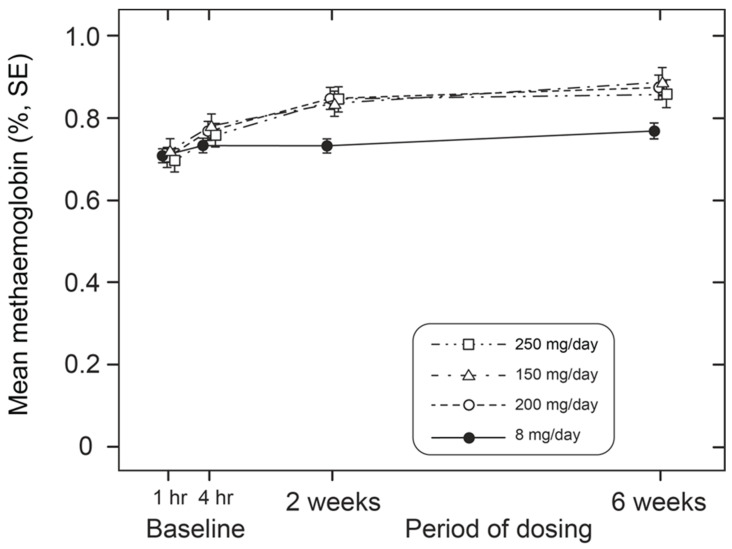
Alterations in metHb levels in patients receiving low (8 mg/day) and high doses (150 mg, 200 mg and 250 mg/day) of HMTM over a 6-week period. Data represent mean (%) ± S.E. Statistical analysis relating to this graph is provided in Table 5.

We next investigated how the effect on hypoxaemia relates to metHb levels measured simultaneously in the same patients. For both treatment groups, there was no consistent difference in metHb levels between baseline and 4 h post-dose (Figure 4, Table 3). Therefore, the acute effect of HMTM on oxygen saturation is independent of any consistent corresponding effect on methaemoglobin at either low or high doses. Over 6 weeks, HMTM at the higher doses (150, 200 and 250 mg/day) systematically increased the metHb levels but not at the 8 mg/day dose (Figure 5, Table 5), although none of the changes in metHb reached statistical significance in this small number of subjects. When the effects on metHb levels were considered in the entire population, the small increases became statistically significant, including at the 8 mg/day dose (Table 5). These results imply that treatment with HMTM is able to improve oxygen saturation while at the same time *increasing* levels of metHb.

### 2.3. Computational Chemistry Model of HMT-Heme Effect

**Figure 6:**
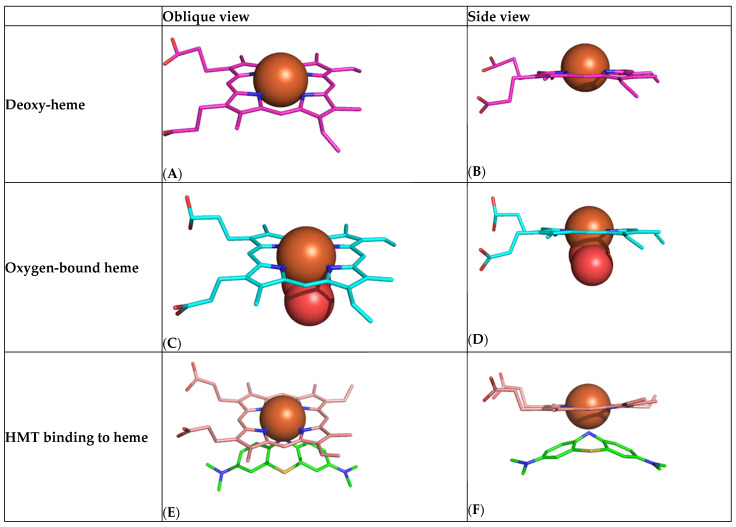
Computational chemistry modelling of the high-affinity HMT/MT^+^–heme interaction. (**A**,**B**) Heme in the nonplanar unbound T-state showing protrusion of the iron atom (brown) above the plane of the porphyrin ring. (**C**,**D**) Heme in the flat oxygen-bound R-state (oxygen shown as two red molecules). (**E**,**F**) Heme adopting the R-state with HMT (green).

We have used computational modelling to study the minimum-energy HMT–heme interaction to better understand how it might be able to produce an increase in oxygen saturation by a mechanism other than reduction in oxidised heme iron (Figure 6). The modelling suggests that HMT is able to bind with high affinity to the heme iron of haemoglobin via the HMT nitrogen in an octahedral geometry and within 2.1 Å of the iron atom (Figure 6). HMT interacts strongly with the $d_{z^{2}}$ and $d_{x^{2}-y^{2}}$ orbitals of the Fe^2+^ electrons, which are oriented at the axial ends and equatorial corners, respectively, of the octahedral complex. We hypothesise that the formation of this HMT–heme complex acts in a manner analogous to the binding of oxygen (Figure 6C,D) in helping to overcome the initial barrier for oxygen binding by facilitating a shift from the T-state (Figure 6A,B) to the R-state (Figure 6E,F). The resulting co-operativity would permit higher relative oxygen saturation to occur at lower PaO_2_ levels, consistent with the implied left-shift in the oxygen–haemoglobin dissociation curve suggested by our analyses.

## 3. Discussion

We report preliminary results of an exploratory analysis of the effects of HMTM treatment on patients who were chronically mildly hypoxaemic at baseline. We show that HMTM is able to enhance oxygen saturation levels in the blood. We investigated 18 subjects who came into either of two Phase 3 trials in mild-to-moderate AD and who had oxygen saturation levels below 94% at baseline, due to a range of incidental chronic cardiorespiratory conditions. We show that single doses of HMTM in the range 4–125 mg were able to increase oxygen saturation levels significantly within 4 h, and that the effect persists over 6 weeks of treatment with the same doses given twice daily. This implies that HMTM is able to bind to haemoglobin in such a way as to enhance oxygen saturation by about 3% in patients with chronic hypoxaemia.

The ability of the MT moiety to correct methaemoglobinaemia is well known. Indeed, MTC is the first-line treatment [2]. As shown by May et al., the MT^+^ of MTC needs to be converted to HMT to permit absorption by red cells, and it is the HMT species which acts to reduce Fe^3+^ to Fe^2+^ in methaemoglobinaemia [18,19]. We have previously reported that there is 20-fold better red cell uptake of the MT moiety when it is administered intravenously as an HMT salt. The present results demonstrate that HMTM administered orally has activity with respect to haemoglobin. We also show that this activity occurs in hypoxaemic patients without pre-existing methaemoglobinaemia and can occur independently of any effect on the level of metHb. Indeed, HMTM at high doses produces small but measurable increases in metHb levels over 4 weeks, despite maintaining the increase in oxygen saturation relative to baseline over this period. Therefore, the effect on oxygen saturation does not depend on the reduction of metHb levels.

We have utilised computation chemistry to understand the anti-hypoxaemia effect of HMT. As both the structure of heme and of HMT are known, it is possible to compute the minimum-energy binding interaction between the two. We show that HMT is able to bind with high affinity within 2.10 Å of the iron atom of haemoglobin by donating a pair of electrons from the central nitrogen of HMT to the $d_{z^{2}}$ and $d_{x^{2}-y^{2}}$ orbitals of the Fe^2+^ electrons. From crystal field theory, this type of interaction has an estimated field factor of 1.2–1.5 [20,21], implying that HMT is able to act as a strong displaceable field ligand. We hypothesise that the formation of this complex facilitates a shift from the T-state in which Fe^2+^ has an ionic radius of 2.06 Å, which is too large to fit into the cavity in the centre of the porphyrin ring [22], to the R-state in which Fe^2+^ has an ionic radius of 1.96 Å, enabling it to fit within the four nitrogen atoms with which it coordinates. Oxygen is able to bind with higher affinity to R-state heme than T-state heme [23], thereby overcoming the initial energy barrier, and subsequent binding of oxygen is further facilitated by cooperativity [24]. This is consistent with a left-shift of the oxygen–haemoglobin dissociation curve [25]. However, the HMT binding is non-optimal compared with oxygen. Whereas the binding distance between the HMT nitrogen and heme iron is 2.10 Å, the corresponding binding distance for oxygen is 1.98 Å. This implies that oxygen would be able to displace HMT when it is available at high pH or low pCO_2_, thereby permitting normal oxygen dissociation to occur with the release of bound oxygen to peripheral tissues. This is consistent with the reduction in respiratory rate observed in severely ill patients treated with the reduced MTC preparation of HMT [17]. The respiratory rate is driven by central and peripheral chemoreceptors sensitive to hypoxia and increased CO_2_ levels which signal tissue hypoxia [26].

The formation of the heme–HMT complex that we describe provides a structural explanation for three different possible effects of the MT moiety on haemoglobin: the reduction Fe^3+^ to Fe^2+^ in methaemoglobinaemia [12], the conversion of Fe^2+^ to Fe^3+^ at high concentrations of MT [27], and the novel effect on oxygen saturation we describe here. In the first two, the formation of the heme–HMT coordinate permits either the donation of an electron or removal of an electron, depending on the availability of adequate levels of NADPH needed to regenerate HMT. This is required to convert the MT^+^ produced from HMT during the reduction of Fe^3+^ back to HMT. At high concentrations of the MT moiety (or in the presence of glucose-6-phosphate dehydrogenase deficiency), the level of MT^+^ exceeds the available reducing capacity of the cell. MT^+^, which is able to form the same co-ordinate with heme via the orientation of the nitrogen atom, oxidises Fe^2+^ to Fe^3+^ and forms HMT. In the case of hypoxaemia occurring in the context of normal red cell physiology, we show that low doses of HMTM are able to improve oxygen saturation with a minimal corresponding effect on levels of metHb. At higher doses of HMTM, there is still enhancement of oxygen saturation, but in addition, Fe^2+^ is oxidised to Fe^3+^.

Since the co-ordination of MT and heme likely provides the same structural explanation in all three cases, it is likely that HMTM at doses less than ~50 mg/day could provide an oral treatment for both hereditary and acquired forms of methaemoglobinaemia and also a range of haemoglobinopathies for which the management of chronic hypoxaemia is a challenge. Similar considerations also apply to the broad range of respiratory or other conditions which likely underly the hypoxaemia detected incidentally in the patients included in the present study. Given the additional evidence of direct antiviral activity of the MT moiety in vitro in cell-free and cell-based models of SARS-CoV-2 [15,28] and other viral infections [29,30], and the promising data reported by Hamidi-Alamdari [17] and colleagues, HMTM could be used in the treatment of both acute COVID-19 and its sequelae. Since the level of blood oxygenation provides the primary determinant of need for mechanical ventilatory support, a drug which combines both antiviral and anti-hypoxaemia benefits would be of considerable interest.

There are several obvious limitations to the present study. The number of patients is small, and the therapeutic benefits were identified incidentally in post hoc analyses, albeit in the context of otherwise well conducted prospective clinical studies. The fact that the SpO_2_ results in the present study are directionally similar to those reported recently by Hamidi-Alamdari [17] and colleagues in a quite different population and using a different preparation of HMT, supports the generalisability of the findings. Based on the available data, we now plan to conduct prospective placebo-controlled studies in larger numbers of patients suffering from conditions in which HMTM could be beneficial. A further limitation relates to the use of standard pulse oximetry which is well documented [31]. The device (Massimo Corporation, model Rad-57) was chosen primarily to monitor metHb levels prospectively, as this is a known dose-dependent side-effect of treatment with both MTC and HMTM [7,8]. A validation study [32] has been reported comparing this device to standard pulse oximetry and metHb was found to have zero bias and a precision of ± 0.45%. Abnormal values of metHb and SpO_2_ were seen in fewer than 1% of over 2000 patients in the HMTM AD trials. The convenience and speed of this method made it preferable to alternatives in our AD trials. The systematic measurement of blood gases is only feasible in more intensive care settings. A further limitation is the lack of direct biochemical evidence for the heme–HMT interaction that we have described the basis of computational chemistry. Further direct biochemical and structural studies of the interaction are required to confirm the hypotheses we have advanced.

### 3.1. Interpretation

Notwithstanding the limitations of the present study, it is intriguing that HMTM is able to produce measurable changes in oxygen saturation. The concomitant changes in metHb that would be expected if a redox reaction were responsible are either absent or are in the wrong direction to explain the elevation in SpO_2_ we report. The computational chemistry modelling that we propose appears to provide a simple structural explanation for three different possible effects of the MT moiety on haemoglobin. Important predictions of the structural model that we propose is that HMTM could provide an oral treatment for hereditary and acquired methaemoglobinaemia in addition to alleviating chronic hypoxaemia in a variety of cardiorespiratory conditions. The availability of an oral treatment with a benign safety profile for use in these conditions would represent a valuable addition to the treatment options currently available.

### 3.2. Take-Home Points

**Study Question:** To determine the effects of oral HMTM on SpO_2_ and metHb levels in patients with mild hypoxaemia not due to COVID-19.

**Results:** Single doses of HMTM in the range 4–125 mg were able to increase oxygen saturation levels significantly within 4 h, and that the effect persists over 6 weeks of treatment with the same doses given twice daily. This implies that HMTM is able to bind to haemoglobin in such a way as to enhance oxygen saturation by about 3% in patients with chronic hypoxaemia.

**Interpretation:** HMTM is able to produce measurable changes in oxygen saturation and has potential as an adjunctive treatment of hypoxaemia. We propose that HMTM could provide an oral treatment for hereditary and acquired methaemoglobinaemia in addition to alleviating chronic hypoxaemia in a variety of cardiorespiratory conditions including COVID-19.

## 4. Materials and Methods

### 4.1. Patients

The study design and results from two Phase 3, double-blind, controlled, randomised, studies of HMTM as a potential treatment for AD were described elsewhere [7,8]. In summary, 890 mild/moderate AD patients were randomly assigned (3:3:4) to 150 mg/day, 250 mg/day or 8 mg/day (intended as a control) for 15 months (clinicaltrials.gov ID- NCT01689246). Similarly, 800 patients with mild AD were randomly assigned to 200 mg/day or 8 mg/day HMTM for 18 months (clinicaltrials.gov ID- NCT01689233). In both trials, MetHb and SpO_2_ levels were measured by pulse oximetry (Massimo Corporation rad 57) at screening, baseline (within 1 h prior to dosing), post-dose during the 4 h observation, and subsequent clinic visits at 2 and 6 weeks.

### 4.2. Estimation of Effect of HMT Treatment on Oxygen–Haemoglobin Dissociation Curve

We have used a standard clinical SpO_2_ to PaO_2_ conversion table (e.g., https://www.acphospitalist.org/archives/2013/11/acph-201311-coding_t1.pdf (accessed on 21 August 2023)) to compare the oxygen–haemoglobin dissociation curves in patients receiving HMTM or those receiving a reduced form of MTC [17] with normal. For patients treated with HMTM, implied PaO_2_ values at baseline and corresponding SpO_2_ values were plotted. After treatment with HMTM, the SpO_2_ values were plotted at the same PaO_2_ values to deduce the implied shift in the dissociation curve. In order to compare these treatment effects with those reported by Hamidi Alamdari and colleagues (2021) [17], SpO_2_ values observed in patients receiving standard of care were used to calculate the implied untreated PaO_2_ values. The SpO_2_ values observed after 5 days of treatment with the reduced MTC preparation were plotted at the same PaO_2_ values to infer the implied shift in oxygen–haemoglobin dissociation.

### 4.3. Statistics

Statistical analyses were performed using R-version 3.5.1, employing paired sample *t*-tests to compare mean SpO_2_ and metHb levels.

### 4.4. Computational Modelling

Quantum chemistry calculations were performed using the self-consistent field (SCF) method within the MOE software suite 2020.09 [33]. SCF calculations used the Austin Model-1 basis set, and geometry optimisations were initially performed using the AMBER 10 force field using extended Hückel theory. The crystal structure of human haemoglobin in the oxy form (2DN1) was used to align the HMT–heme complex [34]. The crystal structure of human haemoglobin in the deoxy form (PDB ID 2DN2) [34] and the crystal structure of human haemoglobin in the carbonmonoxy form (PDB ID 3DN2) [34] were aligned using Pymol.

## Figures and Tables

**Table 1:** Clinical characteristics of patients at baseline.

Characteristic	HMTM Total (*n* = 18)
Age (year)	
Mean (SD)	76.1 (7.8)
Median (range)	77.5 (70–80)
Sex, *n* (%)	
Male	7 (39%)
Female	11 (61%)
Race, *n* (%)	
Black or African American	2 (11%)
White	16 (89%)

**Table 2:** Clinical history of clinical trial subjects presenting with low oxygen saturation. ^a^ Aggravating clinical conditions that may also cause hypoxia if sufficiently severe or chronic. ^b^ Three patients had no predisposing clinical history factors listed.

Subject	Clinical Respiratory/Ventilation Type	Aggravating Clinical Conditions ^a^
1	Sleep apnoea	Hypothyroid/diabetic
2	Insomnia	Often a sign of obstructive sleep apnoea or other mild hypoxia conditions such as paroxysmal nocturnal dyspnoea
3		Hypertension/left bundle branch block (LBBB); left ventricular hypertrophy (LVH)
4	Asbestosis	
5	Oedema	Often a sign of right heart failure or congestive cardiac failure/may also be simple sedentary dependent oedema
6		Hypertension/LBBB/LVH
7		Acute myocardial infarction/hypertension
8		Hypertension
9		Hypertension/coronary artery disease with angioplasty and stent insertion
10 ^b^		
11 ^b^		
12	Asthma (childhood)	
13	Sleep apnoea with uvulectomy	
14	Bronchitis/seasonal allergies/anaemia	
15		Acute myocardial infarction with stent insertion
16 ^b^		
17		Transient ischaemic attack/hypertension
18	Asthma (childhood)/intermittent angioedema/occasional insomnia/pneumonia	Hypertension/hypothyroid/syncope/tachycardia/sepsis

**Table 3:** Change from pre-dose to post-dose oxygen saturation and metHb levels following administration of HMTM at a dose of 4 mg or high doses (75/100/150 mg indicated as 100 mg). Data represent mean (%) ± S.E. Statistical analysis relating to this table are provided in Figure 1 and Figure 4.

	4 mg		100 mg	
	Pre-Dose	Post-Dose	Pre-Dose	Post-Dose
Oxygen saturation, mean % (SE)	91.71 (0.75)	95.43 (0.65)	92.45 (0.28)	95.27 (0.74)
metHb, mean % (SE)	0.70 (0.195)	0.87 (0.198)	0.90 (0.220)	0.65 (0.154)

**Table 4:** Change in oxygen saturation levels in pooled data for patients at baseline (1 and 4 h post-dose) and then after receiving HMTM over a 6-week period. Data represent mean (%) ± S.E.

Visit	Oxygen Saturation	*p*-Value
Baseline (1 h), mean % (SE)	92.167 (0.336)	
Baseline (4 h), mean % (SE)	95.333 (0.505)	0.000139
Week 2, mean % (SE)	95.400 (0.798)	0.0034
Week 6, mean % (SE)	95.571 (0.618)	0.0005

**Table 5:** (A) Change in metHb levels in the subgroup of patients with baseline oxygen saturation levels below 94% receiving HMTM at low (8 mg/day) or high dosages (150/200/250 mg/day) over a 6-week period. (B) Change in metHb levels in all patients with available data receiving HMTM at low (8 mg/day) or high dosages (150/200/250 mg/day) over a 6-week period. Data represent mean (%) ± S.E.

Visit	HMTM Dose (mg/day)			
	8	150	200	250
**A Patients with SpO_2_ < 94% at baseline**				
Baseline, mean % (SE)	0.709 (0.017)	0.721 (0.029)	0.702 (0.025)	0.696 (0.028)
4 h, mean % (SE)	0.733 (0.018)	0.781 (0.030)	0.760 (0.023)	0.758 (0.028)
Week 2, mean % (SE)	0.732 (0.018)	0.835 (0.032)	0.849 (0.027)	0.845 (0.033)
Week 6, mean % (SE)	0.769 (0.019)	0.888 (0.034)	0.873 (0.030)	0.858 (0.033)
**Visit**	**HMTM dose (mg/day)**			
	**8**	**150**	**200**	**250**
**B All patients**				
Baseline, mean % ± SE (N)	0.709 ± 0.017 (754)	0.721 ± 0.029 (268)	0.702 ± 0.025 (398)	0.696 ± 0.028 (265)
4 h, mean % ± SE (N)	0.732 ± 0.017 (754)	0.7811 ± 0.029 (267)	0.760 ± 0.023 (398)	0.758 ± 0.028 (263)
*p*-value	0.09732	0.02212	0.01967	0.005868
Week 2, mean % ± SE (N)	0.733 ± 0.018 (721)	0.831 ± 0.032 (248)	0.849 ± 0.027 (355)	0.837 ± 0.034 (239)
*p*-value	0.1326	<0.0001	<0.0001	<0.0001
Week 6, mean % ± SE (N)	0.770 ± 0.019 (721)	0.889 ± 0.034 (248)	0.873 ± 0.030 (355)	0.851 ± 0.033 (239)
*p*-value	0.0006916	<0.0001	<0.0001	<0.0001

## Data Availability

Data are available from C.M.W. on reasonable request.

## Abbreviations

AD (Alzheimer’s disease), Fe^2+^ (ferrous ion), Fe^3+^ (ferric ion), HMT (hydromethylthionine), HMTM (hydromethylthionine mesylate), MT (methylthionine), MTC (methylthioninium chloride), SCF (self-consistent field), SpO_2_ (blood oxygen saturation).
